# Gelatin/Chitosan Films Incorporated with Curcumin Based on Photodynamic Inactivation Technology for Antibacterial Food Packaging

**DOI:** 10.3390/polym14081600

**Published:** 2022-04-14

**Authors:** Fan Wang, Ronghan Wang, Yingjie Pan, Ming Du, Yong Zhao, Haiquan Liu

**Affiliations:** 1 College of Food Science and Technology, Shanghai Ocean University, Shanghai 201306, China; wangfan970622@163.com (F.W.); wrh13775771822@163.com (R.W.); yjpan@shou.edu.cn (Y.P.); 2 Shanghai Engineering Research Center of Aquatic-Product Processing & Preservation, Shanghai 201306, China; 3 Laboratory of Quality & Safety Risk Assessment for Aquatic Products on Storage and Preservation (Shanghai), Ministry of Agriculture and Rural Affairs, Shanghai 201306, China; 4 Collaborative Innovation Center of Seafood Deep Processing, Dalian Polytechnic University, Dalian 116034, China; duming121@163.com; 5 Engineering Research Center of Food Thermal-Processing Technology, Shanghai Ocean University, Shanghai 201306, China

**Keywords:** gelatin, chitosan, photodynamic inactivation, curcumin, antimicrobial activity

## Abstract

Photodynamic inactivation (PDI) is a new type of non-thermal sterilization technology that combines visible light with photosensitizers to generate a bioactive effect against foodborne pathogenic bacteria. In the present investigation, gelatin (GEL)/chitosan (CS)-based functional films with PDI potency were prepared by incorporating curcumin (Cur) as a photosensitizer. The properties of GEL/CS/Cur (0.025, 0.05, 0.1, 0.2 mmol/L) films were investigated by evaluating the surface morphology, chemical structure, light transmittance, and mechanical properties, as well as the photochemical and thermal stability. The results showed a strong interaction and good compatibility between the molecules present in the GEL/CS/Cur films. The addition of Cur improved different film characteristics, including thickness, mechanical properties, and solubility. More importantly, when Cur was present at a concentration of 0.1 mM, the curcumin-mediated PDI inactivated >4.5 Log CFU/mL (>99.99%) of *Listeria monocytogenes*, *Escherichia coli*, and *Shewanella putrefaciens* after 70 min (15.96 J/cm^2^) of irradiation with blue LED (455 ± 5) nm. Moreover, *Listeria monocytogenes* and *Shewanella putrefaciens* were completely inactivated after 70 min of light exposure when the Cur concentration was 0.2 mM. In contrast, the highest inactivation effect was observed in *Vibrio parahaemolyticus*. This study showed that the inclusion of Cur in the biopolymer-based film transport system in combination with photodynamic activation represents a promising option for the preparation of food packaging films.

## 1. Introduction

In recent years, the increase in the level of consumption escalated the demand for goods and the development of the packaging industry. The world production of packaging materials has increased at an alarming rate of 8% per year [1,2]. However, more than 90% of these materials are discarded. Since most of them are petroleum-based plastic, they are difficult to degrade [3], resulting in serious environmental pollution and destruction of biodiversity. In addition, they may affect human health through water, soil, and air pollution. Therefore, in order to find the perfect substitute, natural polymers, such as proteins, carbohydrates, and lipids, as well as their derivatives, which are biodegradable and edible substances, have been used as packaging materials, attracting substantial interest from researchers in recent decades.

GEL is a promising bio-based natural polymer material. It is made of animal protein derived from the partial denaturation of collagen that widely exists in nature (animal skin, connective tissues or organs, and bones). GEL is a water-soluble molecule with rich biological functional groups, such as hydroxyl, carboxyl, and amino groups, that provide this material with excellent biocompatibility and degradability properties [4]. Therefore, it is widely used in the preparation of bio-based food packaging films. However, the pure GEL film often exhibits poor thermal stability and mechanical properties, due to its high hydrophilicity, which limits the application of the pure GEL film [5]. In order to solve these problems, many studies have improved the functional and physicochemical properties of GEL films by adding polysaccharides, such as chitosan (CS) [6], tara gum [7], carboxymethylcellulose [8], and starch [9]. Among them, GEL/CS is the most widely studied edible composite film that presents good film formation, biocompatibility, biodegradability, and safety [10,11,12,13]. CS, a deacylated derivative of chitin, is the second most abundant alkaline biological polysaccharide, consisting of β-(1–4)-2-acetamido-d-glucose and β-(1–4)-2-amino-d-glucose units. Many studies have reported that CS displays excellent selective gas permeability and good mechanical properties [14,15]. In addition, CS has been widely used as a bio-based food packaging material due to its abundant availability, low cost, and non-toxicity, among other characteristics.

Photodynamic therapy (PDT) presents antitumor, antibacterial, and antivirus effects and is extensively used in clinical treatments in the medical field [16,17,18]. However, Photodynamic inactivation (PDI) has rarely been reported in the food sector. Sterilization methods in the food industry can be divided into thermal sterilization and non-thermal sterilization techniques. Thermal sterilization technology is traditional, widely used, and has a wide sterilization spectrum, but its energy consumption is high, and it may cause food to lose its original nutrition and flavor under high temperature. Non-thermal sterilization technologies, such as irradiation, pulsed electric field, and ultrasound, have limited their application in food, due to expensive equipment and high energy consumption [19,20]. PDI is considered to be a promising microbial control strategy due to its environmental protection, low energy consumption, and low cost [21]. It is expected that this new non-thermal sterilization technology will serve as an auxiliary methodology to solve the new challenges faced by the food industry. The mechanism of PDI is based on three indispensable components, including a light source, photosensitizer, and oxygen. In the presence of blue light (450–460 nm), the photosensitizer is excited to produce reactive oxygen species (ROS), including hydroxyl radical (·OH), and excited state singlet oxygen (^1^O_2_) through electron transfer or energy transfer. These species are able to destroy almost all types of biomolecules (proteins, lipids, and nucleic acids) and, in consequence, may cause bacteria to die [22]. In the present study, Cur was selected as the active photosensitizer. Cur is a natural plant extract containing polyphenolic active substances isolated from the rhizome of the herb turmeric. At present, it is one of the main natural edible pigments worldwide. A large number of clinical trials have evaluated the safety and efficacy of Cur in humans [23,24,25,26], and it has been proven that this pigment presents different functional properties, including anticancer, anti-inflammatory, antioxidant, and antibacterial [27,28,29,30]. Therefore, it is often added to biomedical composites as a functional active substance [31,32].

The purpose of this study was to develop bioactive packaging films with antibacterial properties combined with PDI technology. Moreover, the characterizations of GEL/CS/Cur films with concentrations of Cur (0.025, 0.05, 0.1, and 0.2 mM) were performed by scanning electron microscopy (SEM), thermogravimetric analysis (TGA), moisture content (MC), water solubility (WS) and water vapor permeability (WVP), UV–visible absorption, Fourier-transform infrared (FTIR), and X-ray diffraction (XRD). Furthermore, the effects of the GEL/CS/Cur films against pathogenic bacteria (*E. coli, L. monocytogenes, V. parahaemolyticus*) and spoilage bacteria (*S. putrefaciens*) were investigated in pure culture, using a blue LED system. This novel non-thermal technology has great potential in food packaging and can potentially be used to prevent microbial contamination, extend the shelf-life of products, and ensure food safety.

## 2. Materials and Methods

### 2.1. Materials and Bacterial Strains

GEL (purity ≥ 99%), CS (degree of deacetylation ≥95%; viscosity, 100–200 mpa.s) and Cur (purity >98%) were purchased from Shanghai Macklin Biochemical Co., Ltd. (Shanghai, China). Glycerol was obtained from Shanghai Yuanye Biotechnology Co., Ltd.(Shanghai, China). All other chemicals and solvents were of analytical grade. *Listeria monocytogenes* (ATCC19115 and ATCC7644), *Vibrio parahaemolyticus* (ATCC17802), *Escherichia coli* (ATCC 43895), and *Shewanella putrefaciens* (SP 05 and SP 08) were isolated from salmon in our laboratory and stored at −80 °C, with a glycerol concentration of 50% (*v*/*v*). Cells were activated, isolated, and cultured to obtain a suspension of ~8.0 Log_10_ CFU/mL.

### 2.2. Films Preparation

**Figure 1:**
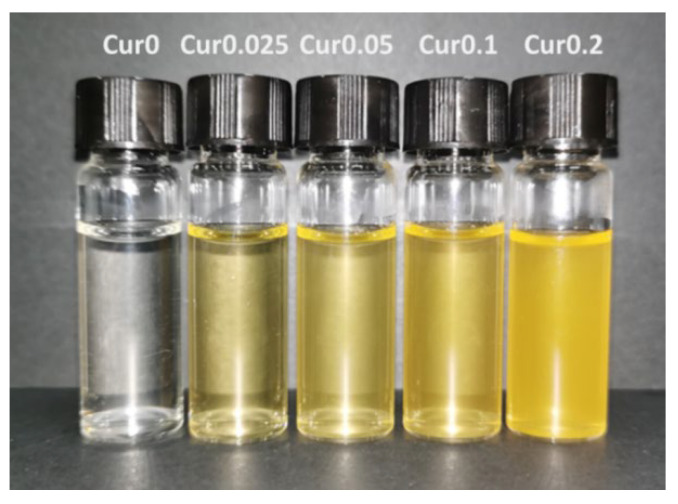
Film-forming solutions (FFSs) with different concentrations of Cur (prepared with a 4:6 mixture of GEL and CS).

In the present investigation, GEL/CS/Cur films were prepared according to the procedures published by Reference [33], but with slight modifications. For this purpose, GEL/CS/Cur film-forming solutions (FFSs) were obtained. In the first step, the GEL FFS (1.5%, *m*/*v*) was prepared by dissolving GEL in deionized water under continuous stirring for 1 h at 55 °C. The CS FFS (1%, *m*/*v*) was obtained by dissolving CS in an acetic acid solution (1% *v*/*v*), under continuous stirring at 55 °C for 2 h. Later, a 0.01 mmol/mL Cur solution was prepared by adding the proper amount of Cur in 95% ethanol. The mixture was stirred at 300 rpm until Cur was dissolved. Subsequently, the GEL FFS and the CS FFS were mixed according to Table 1, and glycerol (0.3%, *m*/*v*) was added. Magnetic stirring was applied for 3 h to achieve homogenization and remove bubbles. Different concentrations of FFSs are shown in Figure 1. Finally, all films were obtained by adding 30 mL of the FFSs to polystyrene Petri dishes (10 cm × 10 cm × 1 cm) and dried in an oven with the air-flow circulation at 40 °C for 24–36 h. Samples were stored at 25 °C and 55% RH in a brown desiccator before further analyses.

### 2.3. Characterizations of the Composite Films

#### 2.3.1. Scanning Electron Microscopy (SEM)

The microstructure of the surface and cross-section of the films was acquired by using SEM (Quanta FEG 250, Hillsboro, OR, USA). The composite films were fixed on a stainless-steel support with a double-sided adhesive, and the analysis was conducted in low vacuum (0.6 Torr), at an acceleration voltage of 20 and 10 kV respectively.

Four bacterial suspensions (1 mL) were centrifuged for 10 min at 4000× *g*. The supernatants were discarded, and the pellets were mixed with 500 μL of glutaraldehyde (2.5%) and formaldehyde (4%) in 0.1 M cacodylate buffer for 8 h at 4 °C. Subsequently the samples were dehydrated in serial dilutions of ethanol solutions (30%, 50%, 70%, 90%, and 100%) for 10 min. The samples were separately placed on the support, with a double-sided adhesive, and coated with gold. The microstructures of cells were observed by using SEM.

#### 2.3.2. Color

Color parameters were measured by using a portable Minolta colorimeter (JZ-300, Osaka, Japan) with a standard white color plate (L_0_ = 99.44, a_0_ = −0.28, b_0_ = 0.54) as the background reference. Results of L* (lightness), a* (red to green), and b* (yellow to blue) were directly read from the colorimeter. The total color difference (∆E) of the films was calculated according to Equation (1) [34]:

(1) $$∆E=\sqrt{{\left(∆L^{*}\right)}^{2}+{\left(∆a^{*}\right)}^{2}+{\left(∆b^{*}\right)}^{2}}$$

where ΔL*, Δa*, and Δb* are the differences between each color value of the standard color plate and film specimen, respectively. Values were expressed as the means of ten measurements on different areas of each film.

#### 2.3.3. UV–Visible Spectra

The UV–Vis transmission spectra of the composite films (1 cm × 4 cm) were obtained in order to evaluate the effect of the addition of Cur on the barrier properties of films to ultraviolet (UV) and visible (Vis) light. Spectra were recorded by using a UV spectrophotometer (UV-3600, Shimadzu, Tokyo, Japan). The film opacity was calculated by using Equation (2) [35]:

(2) $$\mathrm{Opacity}\;\mathrm{value}=\frac{-{\mathrm{logT}}_{600}}{x}$$

where T_600_ is the transmittance at 600 nm, and x is the film thickness (mm).

#### 2.3.4. Thickness and Mechanical Properties

The thickness of film samples was measured by randomly taking the average of 10 points on the film, using a micrometer caliper (Mitutoyo, Japan) with a precision of 0.001 mm. The tensile strength (TS) and elongation at break (EB) of the films were obtained by using an Auto Tensile Tester (XLW-EC, PARAM, Jinan, China). Before testing, film samples were cut into strips (15 mm × 100 mm) and mounted in the tensile grip at an initial distance of 65 mm. Later, samples were stretched at a cross-head speed of 50 mm/min until breaking occurred. At least five replicates were tested for each film.

#### 2.3.5. Moisture Content (MC), Water Solubility (WS), and Water Vapor Permeability (WVP)

The film pieces (2 cm × 2 cm, n = 3, M_1_) were dried in an oven at 105 °C for 24 h to reach a constant weight (M_2_). Samples were then completely immersed in centrifuge tube with 30 mL distilled water. Tubes were shaken at 180 r/min and 26 °C for 24 h. Later, samples were filtered to remove excess water and dried at 105 °C for 24 h until constant weight (M_3_). The MC and WS (%) of film samples were calculated by using Equations (3) and (4) [36].

(3) $$\mathrm{MC}\;\left(\%\right)=\frac{M_{1}-M_{2}}{M_{1}}\times 100$$

(4) $$\mathrm{WS}\%=\frac{M_{2}-M_{3}}{M_{2}}\times 100$$

The water vapor permeability (WVP) of the films was measured gravimetrically according to E96-05 (ASTM, 2005), but with some modifications. In this assay, the glass weighing bottle was filled with 20 mL of distilled water (100% RH). Later, the film sample was placed over the circular opening and sealed tightly with parafilm to prevent the leakage of water vapor. The glass weighing bottles were maintained at a constant temperature of 25 °C. Weight changes of the glass weighing bottles were monitored at intervals of 2 h for a total of 12 h. The slope of weight changes versus time plot was obtained by using linear regression (r^2^ > 0.99). WVTR and WVP were calculated according to Equations (5) and (6) [37].

(5) $$\mathrm{WVTP}=\frac{∆w}{A\times ∆t}$$

(6) $$\mathrm{WVP}=\frac{\mathrm{WVTR}\times L}{∆P\;\times ∆\mathrm{RH}}$$

where ΔW/Δt indicated the weight change as a function of time (g/h), A is the area of the exposed film surface (m^2^), L corresponds to the mean film thickness (m), Δp is the water vapor pressure difference (kPa) between two sides of the film, and ΔRH is the relative humidity gradient across the film (%).

#### 2.3.6. Fourier-Transform Infrared (FTIR) Spectroscopy

Fourier-transform infrared (FTIR) spectroscopy analysis was performed to obtain chemical information of the films’ surface. The spectra of Cur and composite films were obtained by using a spectrophotometer (Thermo IS10, Thermo Fisher, MA, USA) system from 400 to 4000 cm^−1^, with a resolution of 4 cm^−1^ and 32 scans.

#### 2.3.7. X-Ray Diffraction (XRD)

In order to examine the crystalline structure of the films, X-ray diffraction patterns (XRD) were recorded by using a Rigaku Ultima IV X-ray diffractometer (RINT2000, Tokyo, Japan), equipped with a Cu-Kα radiation, at 40 kV voltage and 30 mA current. Samples were scanned over the 2θ range of 5–50°, at a speed of 2°/min (RINT2000, Tokyo, Japan).

#### 2.3.8. Thermogravimetric Analysis (TGA)

The thermal properties of the composite films were determined by using a thermal analyzer (NETZSCH STA 449C, Selb, German). The measured samples were kept in the range of 30–800 °C, and the heating rate was 10 °C/min. Nitrogen was used as the protective gas [38].

### 2.4. Photodynamic Inactivation of the Composite Films

#### 2.4.1. Light-Emitting Diodes (LEDs) System

The blue LEDs (10 W, 450–460 nm, 30 cm) were used as the light source for the photodynamic treatment [39,40]. These LEDs were surrounded by deep photo accessories to prevent the interference of external light sources. The film samples were cut into squares (2 cm × 2 cm) and placed directly on 6-well plates. The distance was adjusted to 5 cm between the light source and film samples. The blue light intensity was 3.8 mW/cm^2^, which was determined by using a PM100D energy meter console (Newton, MA, USA). The obtained energy dosage of each composite film sample was calculated by using Equation (7) [41].

(7) E=Pt

where E = dose (energy density) in J/cm^2^, P = irradiance (power density) in W/cm^2^, and *t* = time in s.

#### 2.4.2. Antimicrobial Activity

The bacterial suspension (100 μL) was evenly distributed on the surface of the GEL/CS/Cur films containing different concentrations of Cur (0, 0.025, 0.05, 0.1, and 0.2 mM). Later, films were exposed to light for 70 min (15.96 J/cm^2^) and then maintained in the dark for another 10 min to ensure that the bacteria were able to attach to the film before irradiation. In addition, GEL/CS/Cur films containing 0.1 mM Cur were irradiated for 30 min (6.84 J/cm^2^), 50 min (11.4 J/cm^2^), 70 min (15.96 J/cm^2^), and 90 min (20.52 J/cm^2^) in order to explore their potential use in PDI of food-borne pathogenic bacteria. After treatment, the films containing bacteria were homogenized with sodium chloride (0.85%, *w*/*v*) for 5 min. The antibacterial effect of the GEL/CS/Cur films was investigated by spreading 100 μL of the suspension onto agar plates and incubated for 12–48 h. Viable cells were quantified as Log CFU/mL. Herein, samples without light treatment and Cur were labeled as negative control (L−C−). In addition, samples with light treatment but without Cur were labeled as illumination control (L+C−). Moreover, samples with Cur but without light treatment were identified as Cur control (L−C+). All the experiments were performed in triplicate.

### 2.5. Statistical Analysis

The experimental data were analyzed by using SPSS (SPSS 17.0 Software, Inc., Chicago, IL, USA). One-way analysis of variance (ANOVA) was used to compare differences between pairs of means (*p* < 0.05).

## 3. Results and Discussion

### 3.1. Optical Properties of Films

Transparency and color of edible food packaging materials are critical properties that influence consumer acceptance, as they directly affect the appearance of the product. The color values (L*, a*, and b*), total color difference (ΔE*), opacity, and picture of the GEL/CS film and GEL/CS /Cur films are shown in Table 2. The L* values of the composite film were in the range of 86.09–93.38, and the brightness of the composite films were not affected by the presence of Cur. However, the a* and b* values of the GEL/CS/Cur composite films were higher than those of the GEL/CS films, which indicated that the yellowness and redness of the composite films significantly improved with the addition of Cur. As a result, the total color difference (ΔE*) of the curcumin-added composite films increased as compared to GEL/CS. Data in Table 2 indicated that the increase in Cur concentration resulted in an increase in film opacity, for which the values were lower than 5 at 600 nm in all cases. Thus, we can conclude that all the films prepared in the present study were transparent. The higher the opacity value, the lower the transparency of the film.

**Figure 2:**
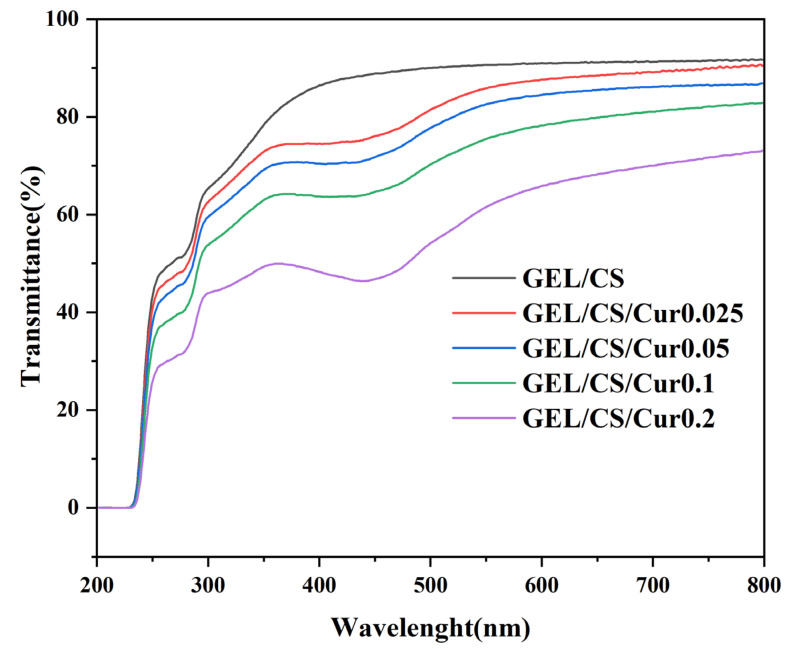
UV–Vis transmittance spectra of GEL/CS and GEL/CS/Cur composite films.

Figure 2 presented the UV–visible light transmission spectra (200–800 nm) of the composite films with and without Cur. The packaging material with good light-barrier properties effectively prevents light transmission and reduces light-induced oxidation of packaged foods, consequently inhibiting the lipid oxidation, nutrient loss, and degradation of active compounds [42]. All composite films exhibited high protection against UV light, which was probably the result of the excellent UV-absorbing properties of aromatic amino acids found in the GEL [43,44]. It was also observed that light absorption by the GEL/CS/Cur composite films decreased in the visible light wavelength region of 390–450 nm. This probably occurred because Cur absorbs light in the range of 400–500 nm, which is similar to the tested wavelength range. Since the phenolic compounds in Cur display excellent light absorption properties, the addition of Cur improved the light-barrier characteristics of the films. With the increase of the Cur concentration, the light transmittance of the composite films decreased. However, all the analyzed films presented good transparency.

### 3.2. Microstructure of Films

**Figure 3:**
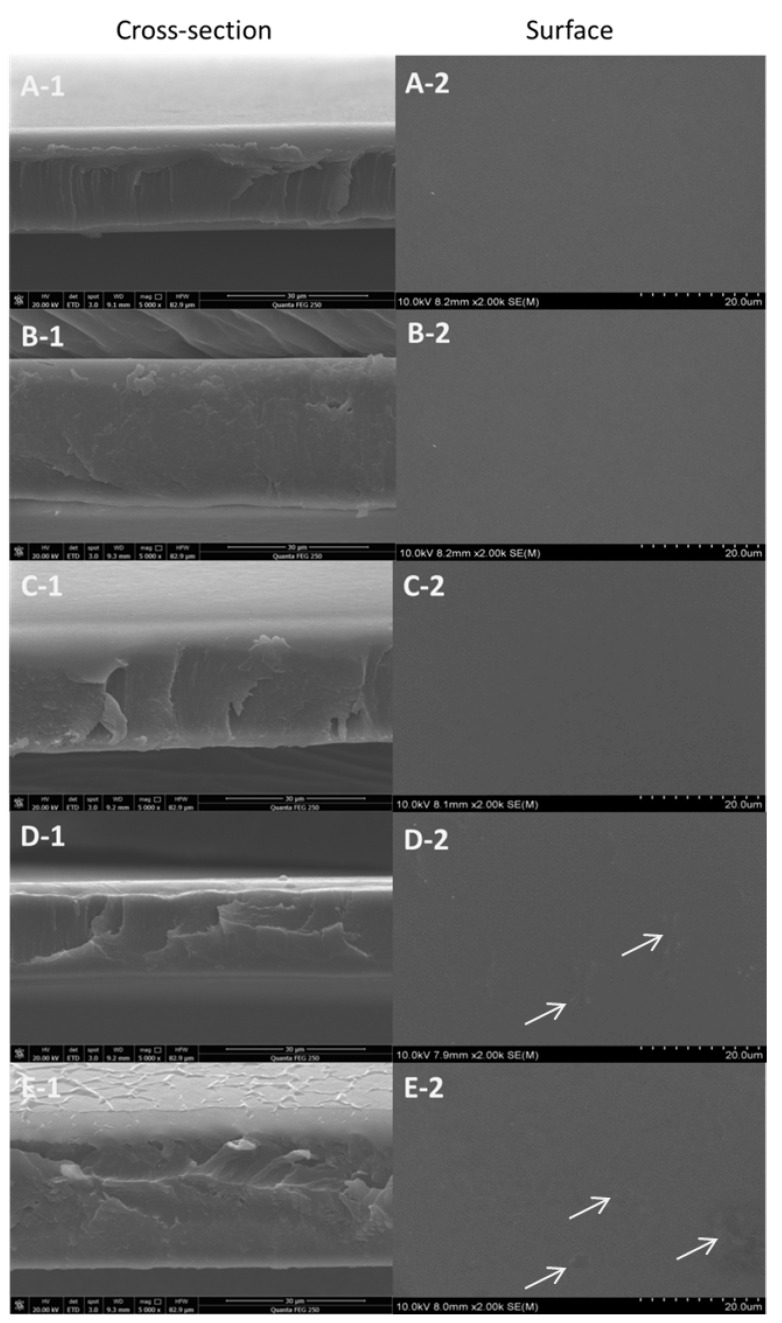
Scanning electron microscopy (SEM) images of the surface (**A-1**–**E-1**) and cross section (**A-2**–**E-2**) of composite films. (**A-1**,**A-2**) GEL/CS films; (**B-1**–**E-1**) and (**B-2**–**E-2**) GEL/CS/Cur films with Cur contents of 0.025, 0.05, 0.1, and 0.2, respectively.

The surface topography and cross-section are used to characterize the microstructure of packaging materials. This information is helpful in determining different properties, including sealing and flexibility. The surface and cross-section of the GEL/CS film and GEL/CS/Cur composite films were observed by using SEM (Figure 3). GEL/CS, GEL/CS/Cur 0.025 and GEL/CS/Cur 0.05 films presented homogeneous and smooth surfaces. However, with increasing Cur concentrations, slight protuberances appeared on the surface of GEL/CS/Cur films. In addition, when films contained Cur, the cross-section of the composite films appeared slightly rough in contrast to the control films. However, the films also displayed a uniform thickness and regular texture. Moreover, no macroscopic phase separation was observed, indicating that Cur was properly dispersed in GEL/CS FFSs. This mainly occurred because of the good materials compatibility. Roy et al. (2017) also found that Cur was well distributed in GEL/Cur composite films [30,45].

### 3.3. Mechanical Properties

Because of the low content of Cur, the thickness of the films varied in the range of 0.33–0.38, with little difference. Data in Table 3 indicate that the tensile strength (TS) and elongation at break (EB) of the composite films increased with respect to controls. The mechanical properties of the fabricated GEL/CS/Cur films were affected by the addition of Cur. It was believed that the hydrogen bond interaction between Cur and GEL/CS was responsible for the improved TS of the films [30]. These results agree with the XRD data. Other studies have reported that EB increases because Cur improves the adhesion between the filler and the polymer [46,47].

### 3.4. Moisture Content (MC), Water Solubility (WS), and Water Vapor Permeability (WVP)

Water sensitivity plays an important role in the wide applications of biodegradable films. The results of the moisture content (MC), water solubility (WS), and water vapor permeability (WVP) of GEL/CS and GEL/CS/Cur films were presented in Table 3.

According to our data, GEL/CS films displayed an MC of 21.96% higher as that quantified in GEL/CS/Cur films. Cur is a hydrophobic molecule with a very small capacity for water retention. This resulted in a continuous MC decrease from 21.29 to 18.54%. The UV–Vis spectra indicated that Cur-containing films absorbed UV–Vis irradiation between 390 and 450 nm, which is in agreement with the maximum absorption of Cur that occurs between 400 and 500 nm. As the Cur concentration gradually increased, the maximum absorption shifted to blue. This explains why the WS of GEL/CS/Cur films increased by increasing the concentration of Cur. Gómez-Estaca et al. [48] also observed a blue shift in the absorption spectrum of Cur, which occurred because of the formation of a Cur–GEL complex. This complex is responsible for the increase in Cur solubility in water. In addition, the increase in WS could be explained by the fact that the GEL swell in water and was partially soluble at 25 °C. As a result, with the increase in Cur content, the WS value of the GEL/CS/Cur films exhibited a continuous increase, reaching values between 20.46 and 24.75%.

The water barrier properties meet the requirements for food preservation. Therefore, a lower WVP should be considered in the application of biodegradable films. In the present study, by increasing the concentration of Cur, the overall value of WVP increased, with small differences. The complex composition of polymers results in weak cohesion between their components, leading to a less disordered crystal structure [49]. This phenomenon was the possible cause of the WVP increase.

### 3.5. Physicochemical and Structural Properties of Films

**Figure 4:**
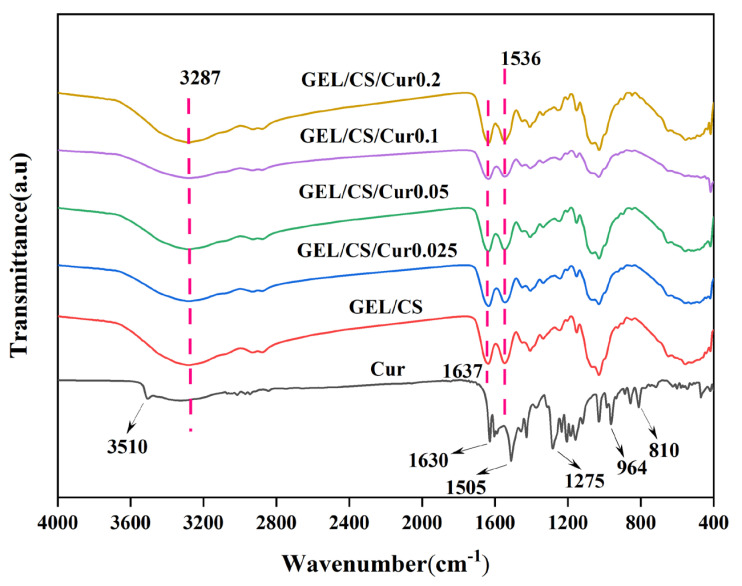
Fourier–transform infrared (FTIR) spectra of GEL/CS and GEL/CS/Cur composite films.

The chemical nature of the interactions in the GEL/CS/Cur films were investigated by using FTIR (Figure 4). The Cur spectra showed a peak at 3510 cm^−1^, which was attributed to the phenol O-H stretching vibrations. Additional peaks at 1630 and 1505 cm^−1^, corresponding to C=O and C=C stretching vibrations of the Cur structures, were also observed. Furthermore, peaks at 1275 cm^−1^ referred to ether C-O stretching vibration, and those at 810 and 964 cm^−1^ represented the C-H bending vibration, which was consistent with the alkene structure in Cur [30]. In the GEL/CS films, a broad band at 3287 cm^−1^ was attributed to the overlapped −OH stretching vibration of CS and GEL. The peak at 1536 cm^−1^ corresponded to the amide II C=O stretching vibration of CS, which overlapped with that of GEL [6]. Moreover, the spectra of GEL/CS showed bands corresponding to different amide types present in GEL (A, B, I, II, and III). These peaks were observed at 3285, 2936, 1637, 1541, and 1242 cm^−1^, respectively, which indicated the existence of N-H, −CH_2_, and C=O stretching vibration, with N-H bending coupled to C-N stretching, and C-N stretching coupled to N-H bending [50]. Interestingly, after adding different Cur concentrations during the preparation of the GEL/CS/Cur composite films, the spectra of these materials displayed similar main peaks as those of the GEL/CS films. This indicated that the addition of Cur preserved the chemical structures originally present in GEL/CS films. Thus, the addition of Cur did not produce new chemical structures [51]. Moreover, changes in peak intensities were the result of physical interactions, non-covalent interactions, and hydrogen bonding [52,53].

**Figure 5:**
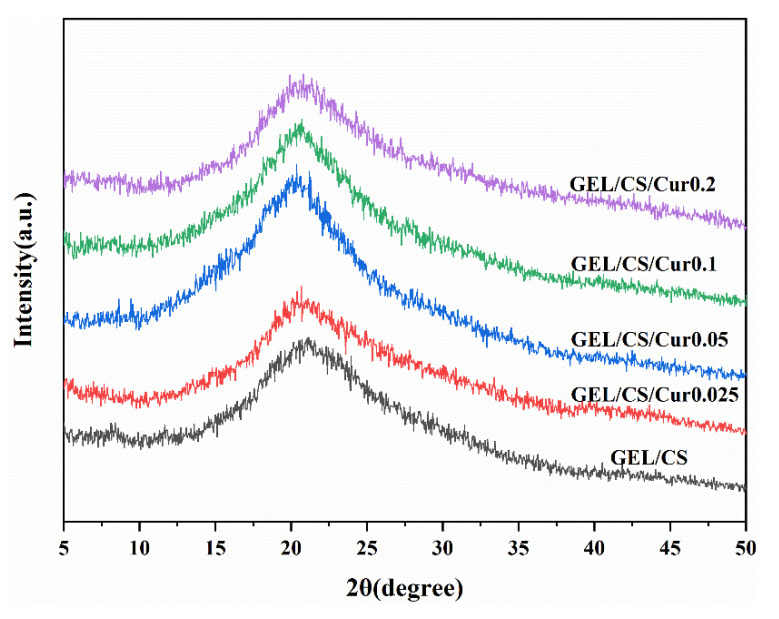
X-ray diffraction patterns of GEL/CS and GEL/CS/Cur composite films.

The crystalline structures of the composite films were discussed by XRD analysis (Figure 5). All composite films presented a similar wide diffraction peak at 2θ = 20°, which was considered to be the random coiled conformation of GEL, and this meant that the film with Cur added was amorphous [54]. The XRD data of the GEL/CS/Cur composite films showed that the Cur addition resulted in an increased crystallinity. For this reason, the diffraction peak intensity increased [55]. However, according to the literature [56], pure Cur was a crystalline material that presented a series of diffraction peaks between 7° and 30°. The disappearance of Cur peaks in the GEL/CS/Cur XRD pattern probably occurred because Cur was present at low concentrations. Thus, the characteristic peaks of GEL/CS overlapped with that of Cur [46].

### 3.6. Thermal Properties

**Figure 6:**
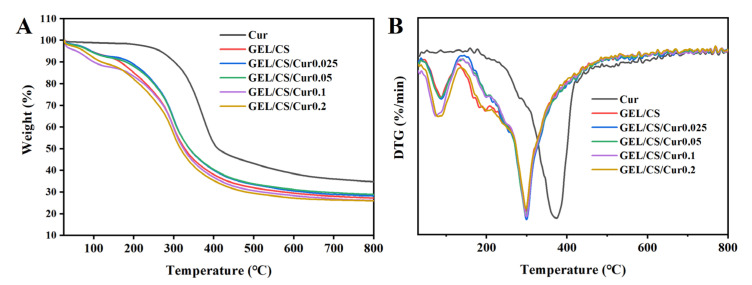
Thermogravimetric (TG) (**A**) and derivative thermogravimetric (DTG) (**B**) thermograms of Cur powder, GEL/CS, and GEL/CS/Cur composite films.

The thermal stability of the composite films was investigated by using thermogravimetric analysis (TGA) and derivative thermogravimetric analysis (DTG), and the results are shown in Figure 6. Cur is a hydrophobic molecule with a very low capacity for water retention and is stable until 200 °C. After this point, Cur is continuously degraded before 450 °C. However, it reaches the maximum degradation rate at 375 °C, and the residue rate was 35% at 800 °C. These results were consistent with the reported literature [48].

The weight loss rates of the GEL/CS/Cur films were faster than those of Cur and showed the similar weight-loss characteristics of the GEL/CS films. The weight-loss process was divided into two main stages: (1) water loss in the range between 50 and 150 °C, and (2) degradation of composites at 200–800 °C [57]. The maximum degradation rate occurred at about 300 °C, and the rapid degradation of Cur disappeared at 375 °C. In addition, the decomposition temperature for Cur was higher than that for composite films. This proved that Cur had good compatibility with GEL and CS, and that Cur was well embedded in GEL/CS films, forming a uniform system [58]. When the Cur was added at a 0.05 mM concentration, the residual rate of the film was the highest one (32%), which also indicated that the thermal stability of GEL/CS/Cur film was improved [57].

### 3.7. In Vitro Antimicrobial Properties

**Figure 7:**
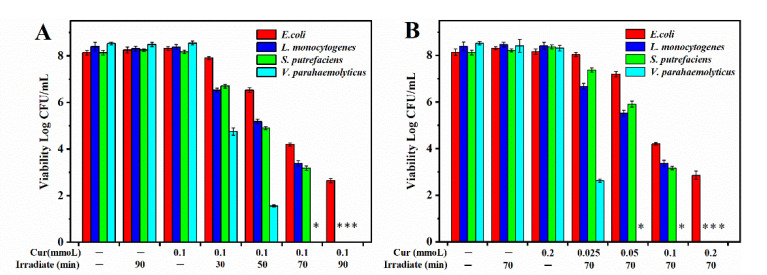
Antibacterial effect of the PDI-treated composite films against *E. coli*, *L. monocytogenes*, *S. putrefaciens*, and *V. parahaemolyticus* at different illumination times (**A**) and Cur concentrations (**B**). * Indicates absence of growth.

**Figure 8:**
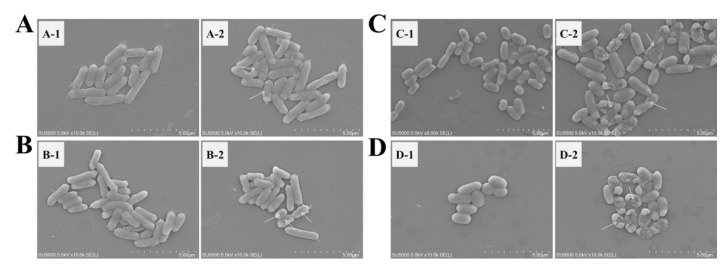
Effects of the curcumin-medicated PDI on the outer membranes of the four bacterial cells. (**A**–**D**) *E. coli*, *L. monocytogenes*, *S. putrefaciens*, and *V. parahaemolyticus*, respectively. (**A-1**–**D-1**) L−C−; (**A-2**–**D-2**) 15.96 J/cm^2^ and 0.1 mM Cur.

In the present research, we evaluated the antibacterial potency of the composite films against *E. coli*, *L. monocytogenes*, *V. parahaemolyticus*, and *S. putrefaciens* (Figure 7 and Figure 8). For this purpose, we selected blue LED illumination and Cur concentration as study variables. As it was shown in Figure 7, individual LED illumination (L+C−) and Cur treatment (L−C+) did not cause significant changes in the antimicrobial activity of the films as compared to the negative control (L−C−).

When the illumination time was increased, the irradiation dose increased and the antibacterial activity improved. These results were shown in Figure 7A. Obviously, the fabricated GEL/CS/Cur films exhibited good antibacterial activity when the Cur concentration was treated by 0.1 mM after 30 min of irradiation (6.84 J/cm^2^). In the case of *V. parahaemolyticus* cells, a decrease from 8.55 to 4.76 Log CFU/mL was observed. Moreover, after the GEL/CS/Cur film with 0.1 mM Cur was irradiated for 70 min (15.96 J/cm^2^), no bacterial cells were detected. Furthermore, *L. monocytogenes* and *S. putrefaciens* cells were killed and could not be detected after 90 min of irradiation (20.52 J/cm^2^). It was also observed that, when the PDI illumination time increased from 30 min (6.84 J/cm^2^) to 90 min (20.52 J/cm^2^), *E. coli* cells presented a continuous decrease from 8.15 to 2.64 Log CFU/mL.

The effects of the Cur concentration on the inactivation of the four bacterial cells were evaluated in Figure 7B. In all cases, an increase in Cur concentration from 0.025 to 0.2 mM led to a significant decrease in the number of bacterial cells. Among the four species, *V. parahaemolyticus* was the most affected, as the number of cells decreased to 2.63 Log CFU/mL after 70 min of irradiation (15.96 J/cm^2^). When Cur concentration in the GEL/CS/Cur films increased to 0.05 mM, none of *V. parahaemolyticus* cells was detected. After 70 min of irradiation with 0.1 mM Cur, *L. monocytogenes* and *S. putrefaciens* cells decreased to 3.38 and 3.17 Log CFU/mL, respectively. Likewise, none of the bacterial cells was detectable when the Cur concentration increased to 0.2 mM. In the case of *E. coli*, when the Cur concentration augmented from 0.025 to 0.2 mM, the number of cells decreased from 8.15 to 3.02 Log CFU/mL.

In the negative control (L−C−), four bacterial cells were plump and rod-shaped (Figure 8). Under the PDI treatment with the illumination time of 70 min (15.96 J/cm^2^) and the Cur concentration of 0.1 mM, the morphological deformation and groove appeared in the *E. coli*, *L. monocytogenes*, and *S. putrefaciens* cells, and the cell surfaces of *S. putrefaciens* and *V. parahaemolyticus* appeared atrophied and ruptured. Obviously, after PDI treatment, *V. parahaemolyticus* cells were most seriously damaged.

Based on the obtained results, it was concluded that the PDI-induced antimicrobial films displayed a typical Cur concentration and irradiation dosage-dependent feature. The photosensitizer Cur produced singlet oxygen and some reactive oxygen species when exposed to blue light (450–460 nm) irradiation, which presents strong oxidative effects and is able to destroy different macromolecular structures, such as proteins, DNA, and lipids. This process may result in the destruction and even death of cells and, in consequence, can be used to eliminate pathogenic bacteria. Previous studies have reported that PDI-induced antimicrobial films exhibited a broad-spectrum antibacterial activity [27,59].

## 4. Conclusions

Degradable and environmentally friendly GEL/CS/Cur composite films were prepared via solution casting. As the photosensitizer of PDI technology, Cur was uniformly dispersed in the fabricated films. Cur presented a high compatibility with GEL and CS. For this reason, this compound improved the mechanical properties and thermal stability of the films. Moreover, low Cur concentrations contributed to smooth the continuous surfaces. However, the MC value decreased and WVP slightly changed, suggesting that the water barrier property of the film was enhanced. The resulting films were resistant to UV and visible light. We would also like to emphasize the fact that the PDI-mediated GEL/CS/Cur films presented a great antibacterial effect in regard to *E. coli*, *L. monocytogenes*, *V. parahaemolyticus*, and *S. putrefaciens* in pure cultures. Furthermore, with the increase in Cur concentration and illumination time, the inactivation effect was enhanced. According to our results, we believe that edible composite films combined with Cur have a great potential application in PDI technology for microbial control in the food packaging industry.

## Figures and Tables

**Table 1:** Film nomenclature and final formulation of film-forming dispersions.

Film Nomenclature	Gelatin (%, *w*/*v*)	Chitosan (%, *w*/*v*)	Ratio (%, *w*/*v*)	Glycerol (%, *w*/*v*)	Curcumin (mmol/L)
GEL/CS	1.5	1	4:6	0.3	-
GEL/CS /Cur0.025	1.5	1	4:6	0.3	0.025
GEL/CS /Cur0.05	1.5	1	4:6	0.3	0.05
GEL/CS /Cur0.1	1.5	1	4:6	0.3	0.1
GEL/CS /Cur 0.2	1.5	1	4:6	0.3	0.2

**Table 2:** Color parameter (L*, a*, b*, and ΔE*; n = 10), opacity (n = 3) values and digital images of the composite films.

Film Samples	L *	a *	b *	∆E	Opacity	Image
GEL/CS	93.38 ± 0.07 ^a^	0.04 ± 0.03 ^d^	3.23 ± 0.82 ^c^	4.13 ± 0.46 ^c^	1.22 ± 0.04 ^e^	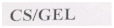
GEL/CS /Cur0.025	90.73 ± 0.47 ^b^	1.23 ± 0.26 ^c^	15.00 ± 2.12 ^b^	15.64 ± 1.95 ^b^	1.73 ± 0.06 ^d^	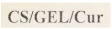
GEL/CS /Cur0.05	89.91 ± 0.20 ^bc^	1.69 ± 0.10 ^bc^	16.63 ± 0.39 ^b^	17.47 ± 0.43 ^b^	2.09 ± 0.07 ^c^	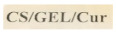
GEL/CS /Cur0.1	89.45 ± 0.41 ^c^	2.22 ± 0.43 ^b^	17.80 ± 2.03 ^b^	18.79 ± 2.07 ^b^	2.83 ± 0.07 ^b^	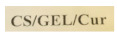
GEL/CS /Cur 0.2	86.09 ± 0.85 ^d^	6.47 ± 0.63 ^a^	30.76 ± 3.03 ^a^	32.65 ± 3.17 ^a^	4.69 ± 0.06 ^a^	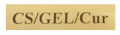

^a,b,c,d,e^ Different superscript letters between columns indicate significant difference between the results (*p* < 0.05) (ANOVA).

**Table 3:** Thickness, tensile strength (TS), elongation at break (EB), moisture content (MC), water solubility (WS), and water vapor permeability (WVP) of the GEL/CS films and those GEL/CS/Cur films with different concentrations of Cur.

	Thickness (μm)	TS (MPa)	EB (%)	MC (%)	WS (%)	WVP (g·mm/m^2^·h·kPa)
GEL/CS	0.33 ± 0.06 ^b^	14.12 ± 0.57 ^e^	53.19 ± 1.27 ^e^	21.96 ± 0.42 ^a^	20.46 ± 1.53 ^d^	0.304 ± 0.029 ^ab^
GEL/CS/Cur0.025	0.34 ± 0.05 ^b^	14.74 ± 0.44 ^d^	56.01 ± 3.15 ^d^	21.29 ± 0.56 ^b^	22.06 ± 0.57 ^c^	0.296 ± 0.013 ^ab^
GEL/CS/Cur0.05	0.36 ± 0.01 ^a^	15.43 ± 0.66 ^c^	59.08 ± 1.40 ^c^	20.04 ± 0.11 ^c^	22.33 ± 1.16 ^c^	0.289 ± 0.008 ^b^
GEL/CS/Cur0.1	0.37 ± 0.02 ^a^	16.85 ± 0.45 ^b^	60.72 ± 1.59 ^b^	19.38 ± 0.73 ^d^	23.42 ± 0.63 ^b^	0.319 ± 0.026 ^ab^
GEL/CS/Cur0.2	0.38 ± 0.02 ^a^	18.12 ± 0.31 ^a^	65.26 ± 0.62 ^a^	18.54 ± 1.12 ^e^	24.75 ± 0.73 ^a^	0.325 ± 0.014 ^a^

Reported values for each film are means ± standard deviation (n = 10 for thickness; n = 3 for TS, EB, MC, WS, and WVP). ^a,b,c,d,e^ Different superscript letters in the same column indicate significant differences between samples (*p* < 0.05), according to ANOVA.

## Data Availability

Not applicable.
